# The impact of worksite interventions promoting healthier food and/or physical activity habits among employees working ‘around the clock’ hours: a systematic review

**DOI:** 10.29219/fnr.v62.1115

**Published:** 2018-08-02

**Authors:** Anne Dahl Lassen, Sisse Fagt, Maria Lennernäs, Maria Nyberg, Irja Haapalar, Anne V. Thorsen, Anna C. M. Møbjerg, Anne M. Beck

**Affiliations:** 1 Division for Risk Assessment and Nutrition, Technical University of Denmark, Kemitorvet, Lyngby, Denmark; 2 Department of Occupational and Public Health Science, University of Gävle, Gävle, Sweden; 3 Department of Food and Meal Science, Kristianstad University, Kristianstad, Sweden; 4 School of Social and Political Sciences, The University of Melbourne, Melbourne, Victoria, Australia; 5 School of Applied Educational Sciences and Teacher Education, Savonlinna, Finland; 6 Institute for Nursing and Nutrition, University College Copenhagen, Copenhagen N, Denmark; 7 Clinical Nutrition Research Unit, Copenhagen University Hospital Herlev-Gentofte, Gentofte, Denmark

**Keywords:** public health, health promotion, occupational health, shift work nutrition, participatory and empowerment strategies

## Abstract

We conducted a systematic review of randomised studies on the impact of worksite interventions to promote healthier food and/or physical activity among people who work irregular hours ‘around the clock’, that is, outside of ordinary daytime working hours. The population–intervention–comparator–outcomes–study (PICOS) design format was used. Data sources were PubMed and CINAHL. An updated search was conducted on October 2017 using Google Scholar and the related articles function in PubMed on initially included studies to identify additional studies. Risk of bias was used to assess study quality. A total of seven studies (reports published in 14 papers) were included in the systematic review: Two interventions with a broader lifestyle approach, three focusing on physical exercise and two on providing healthier food or meal options. The studies had sample sizes from 30 to 1,000 and targeted a mixture of occupations, including both male- and female-dominated occupational groups. The interventions lasted from 2 to 12 months. Only one had an extended follow-up. In general, the studies showed small-to-moderate effect sizes on several measures, including dietary and/or physical activity measures, suggesting acceptable effectiveness for interventions involving community-level behaviour change. Our findings highlight a need to further develop and implement well-designed health promotion interventions with comparable outcome measures and effect size reports. A mixture of health promotion strategies is recommended for future practice in this target population, including individually tailored programmes, improving the food and physical activity environment and using broader lifestyle approaches including the use of participatory and empowerment strategies. While more research is needed in this field, the existing knowledge base on effective approaches awaits translation into practice.

The workplace has been identified as an important setting in which behavioural patterns, including healthy eating, physical activity as well as sleep hygiene, can be promoted (1, 2). Worksites provide a natural social context and could potentially reach a large number of people, including many who would otherwise be unlikely to engage in preventive health behaviour (3, 4). Workers’ good health and well-being is vital for workplace competitiveness and productivity, a long life and a high quality of life (5). This is also emphasised in the EU Public Health Programme for 2014–2020 (6), but there appears to be a gap between political intentions and implementation (4, 7–9).

The political agenda is taking place in a context that has been created by major changes in working life. The modern society has become a ‘24-h society’ in which people can buy goods, including food, and go to restaurants ‘around the clock’. An increasing amount of people are employed in shift work or working outside the ordinary daytime working period and therefore required to work and eat their meals at unconventional hours (10, 11). As the shift work pattern may vary depending on the time of day, the rotation cycle and the direction of rotation of shifts, and may also involve a mobile working place, it creates additional challenges to the food situation at work (12). While there is no consensus definition of the term ‘shift work’ in the published scientific literature, it is often referred to as work conducted primarily outside of ordinary daytime working hours and a pattern of shifts that may be permanent or rotating (13).

Approximately 21% of the workforce in Europe participate in shift work (10) which in this review is referred to as working irregular hours or extended hours ‘around the clock’. Occupations that would fall into this category can be found within the health care sector, manufacturing sector, retail and service sectors. Previous research has linked shift work and working irregular hours to reduced well-being, increased health risks, metabolic syndrome and obesity (13–15), and to poorer eating habits, including a higher energy intake among shift workers compared with day workers (16). However, it has been suggested that it is the timing of meals and eating occasions, rather than the dietary composition that differs between day and shift workers (17–19). Circadian stress due to eating and sleeping in the wrong phase of inherent circadian rhythms is believed to be a main contributor to metabolic disorders in shift workers (20, 21).

Factors that may negatively affect workers’ ability to make healthy food and physical activity choices during working hours include a lack of workers’ influence on work organisation and hours worked, lack of social support and feelings of lack of personal agency and control over the job situation. In addition to the negative effects caused by circadian stress, health risks may be increased by the fact that employees working irregular hours often have limited access to healthy meals and snacks at work (22, 23) and that they are not included in workplace health-promoting activities and initiatives to the same extent as workers in day jobs (24).

In line with the adoption of ecological and socio-ecological models in health promotion, a change has been seen towards moving nutrition from a primarily individual issue to an environmental concern addressing both the physical and psychosocial work environment (3, 14, 25). Accordingly, the use of participatory and empowerment strategies has become important in assuring programme responsiveness to employees’ needs and priorities (3, 26). Research evidence on the effectiveness of different health promotion strategies, including educational, environmental and/or multi-component strategies, in worksites during ordinary daytime working hours suggests that these may be effective in improving dietary habits (27, 28), increasing physical activity (29), reducing body weight (30–32) and increasing work productivity (33). However, uncertainty exists especially regarding the effectiveness and feasibility of health promotion interventions among the working population working ‘around the clock’.

The aim of this study was to conduct a systematic review of randomised studies on the impact of worksite interventions to promote healthier food and/or physical activity among people who work ‘around the clock’.

## Material and methods

A systematic review was undertaken and reported according to the guidelines of the PRISMA statement (34). The protocol was registered with Prospero (registration number CRD42016045216).

### Eligibility criteria

Criteria for study inclusion were developed using the population–intervention–comparator–outcomes–study (PICOS) design format. The resulting PICOS can be found in Table 1, where ‘population’ included people working irregular hours (e.g. shift workers in permanent 2-shift work, permanent night work or 3-shift including night work) or extended working hours (e.g. 12- or 24-h shifts) and ‘intervention’ included studies that focused on developing a healthy working environment defined as interventions to improve dietary habits and/or increase physical activity for a month or more.

**Table 1:** Study eligibility criteria according to PICOS

Author/year	N	Sample	Shift system	Intervention	Length	Design	Critical/important outcome measures (Tool) Significant effects highlighted in bold	Risk of bias †)
Leedo et al. 2017 (51)	60 M/F	Hospital nurses/nursing-aides/physicians (Denmark) Mean age: 45.1 ± 9.3 years Mean BMI: 24.1 ± 3.5	Day time workers and shift workers	**Change of meal offer:** Water, healthy snacks and healthy cold meals during each shift versus own lunch	8 weeks	Cross-over	Anthropometrics (BMI, weight) Reaction time (Go/No-Go test) **Profile of Mood States (POMS) (shift workers)** **Dietary intake (dietary record)**	Low within the larger study. High within the shift workers subsample
Matsugaki et al. 2017 (52)	30 F	Hospital nurses (Japan) Age: 20–40 years BMI: app. 20	Shift work, full time	**Physical exercise:** Supervised versus non-supervised resistance and aerobic training two sessions/week	12 weeks	RCT	**VO_2_ max (cycle)** Muscle strength (knee extension) Anthropometrics (BMI) Body composition (body fat, muscle mass) Blood pressure Pulse rate Blood measurements (**cholesterol**, glucose, oxidative stress) Depression (BDI-II) Profile of Mood States (POMS)	Unclear
Härmä et al. 1988 1988 (39, 40)	75 F	Hospital nurses/Nursing-aides, (Finland) Age: 2 0–49 years; BMI?	38 h/week, irregular rotation of 8–10 h day, evening and night shifts	**Physical exercise**: Training program targeting circulatory and muscular systems (jogging, running, swimming, skiing, walking and gymnastics); 2–6 ×/week, 60–70% maximum heart rate versus usual activity	4 months	2-arm RCT (2:1)	**VO_2_ max (cycle)** **Strength (sit-ups/30s)** **Resting heart rate** Weight Body composition (skinfolds mm) Subjective Sleep (Diary) **Fatigue (questionnaire)** **Sleep Length (h)** Sleep Quality (questionnaire) Body temperature **Alertness (VAS)** Short term memory (SAM-test) **Muscular, GI** and nervous symptoms	High
Morgan et al. 2011 and Morgan et al. 2012 (POWER) (41, 42)	110 M	Overweight/obese aluminium plant workers (Australia) Mean age: 44.4 ± 8.6 years; BMI 30.5 ± 3.6 (45.5% obese)	Four shifts (schedule not reported)	**Lifestyle intervention**: Group-based intervention for weight loss based on Social Cognitive Theory; one-on-one information session, study website, resource booklet, pedometer and financial incentive versus usual activity	14 weeks	Cluster randomised	**Weight (loss)** **Waist circumference** BMI Blood pressure (**systolic)** **Resting heart rate** Physical habits, dietary habits, healthy eating practices, dietary stage of change, self-efficacy *) (questionnaire) Sleepiness during day (Epworth scale) Quality of life *) (SF-12) **Workplace productivity (WLQ)** **Injuries at work** **Absenteeism**	Low
Guillermard et al. 2010 (43)	1,000 M/F	Worker in factory, nurses, firefighters, police officer, other (France) Age: app. 32 years BMI: app. 24	2-shift work or 3-shift work (83%)	**Change of meal offer:** Fermented dairy product containing lactobacillus casei versus placebo to drink (100 g) 2 times per day	3 months (1 month follow-up)	2-arm RCT	**Common infectious diseases** (CID) (upper tract, lower tract, GI) (diary + medical examination + pathogens) **Immune parameters** Duration, **Days with fever** Sick leave Medication (prescribed/self-medication) Quality of life (SF-36) Adverse events (BP, heart rate, weight)	Low
Lim et al. 2015 (44)	30 M	Type of work? (South Korea) Age: app. 57 years BMI: app. 23	Night shift	**Physical exercise:** 3 days walking exercise per week (60–79% VO_2_ max) 3 × 10 min/day versus usual activity	10 weeks	2-arm RCT §)	**Weight,** BMI **§)** Body composition (LBM, **% fat**) Blood pressure **Biomarkers** (cathepsins)	High
Elliot et al. 2004 (PHLAME pilot) (50)	33 M?	Fire fighters (USA) Age: app. 44 years BMI: app. 28	24 h works followed by 48 h off duty	**Lifestyle intervention:** Team-based curriculum (model 1), individual counsellor meetings (model 2) versus usual care Goals: Increase physical activity, servings of fruit and vegetables, reduce fat, improve energy balance versus information	6 months	Cluster randomised §)	**Cholesterol** (blood) BMI, Weight. Body composition (skinfold, % fat) **Dietary habits, exercise habits, mediating aspects** (questionnaire) Peak oxygen uptake (treadmill)	High
Kuehl et al. 2005 (46) Elliot et al. 2007 (45) Ranby et al. 2011 (48) MacKinnon et al. 2010 (PHLAME) (47)	599 (397) 97% M	Fire fighters (USA) Age: 41 ± 9 years BMI: app. 27	24 h works followed by 48 h off duty	**Lifestyle intervention:** Team-based curriculum (model 1), individual counsellor meetings (model 2) versus usual care Goals: Increase physical activity, servings of fruit and vegetables, reduce fat, improve energy balance versus information	12 months and 4 years follow-up	Cluster randomised	12 months **BMI, Weight** Body composition (skinfold, mm) Dietary habits*), exercise habits, **well-being** (questionnaire) Peak oxygen uptake (treadmill) Strength (**sit-ups** and sit and reach) **Work injuries** **Days of** **Injury claims** 4 years’ follow-up Weight Dietary habits, exercise habits, well-being (questionnaire) Peak oxygen uptake (treadmill)	High
Kuehl et al. 2013 (PHLAME) (49)	1,369 93% M	Fire fighters (USA) No data about age or BMI	24 h works followed by 48 h off duty	**Lifestyle intervention** Team-based curriculum (model 1), individual counsellor meetings (model 2) versus usual care Goals: Increase physical activity, servings of fruit and vegetables, reduce fat, improve energy balance versus information	7 year period	Retrospective data before after intervention comparison	**Compensation claims** Medical costs	High ‡)

M = male, F = female, BMI = body mass index, LBM = lean body mass.

* Some of the parameters are significant; † see text for explanation; † since data are based on the PHLAME study with a high risk of bias; § data different at baseline.

The following studies were excluded: interventions conducted among non-shift workers (i.e. workers working ordinary office hours); interventions conducted among workers with extreme work schedules or workers who cross time zones (e.g. astronauts and air crew); interventions conducted in simulated work environments and conditions; literature reviews, commentaries, editorials, opinion pieces, policy documents, consensus statements, study protocols; lacking both pre- and post-intervention critical outcome measures; interventions that were designed to improve profit or turnover; and interventions that were reported in a language other than English.

Critical outcome measures focused on dietary and physical activity. Important outcomes were measures for general well-being, quality of life, sleep circadian rhythm, cognitive performance, mood, psychological stress, blood measurements, body composition, muscle strength, influence on work (e.g. productivity, absenteeism, use of medication, work injuries and medical costs), adverse events and dropouts. Outcomes at both pre- and post-interventions were included, as well as outcomes at follow-up, if reported.

### Search strategy

Relevant studies were identified by searching two different electronic databases: PubMed and CINAHL. There were no time restrictions. Date of search was 14 June 2016. A full example of the search terms used in PubMed is outlined in Appendix A. An updated search was conducted on 18 October 2017 by performing a search on Google Scholar. We also used the related articles function in PubMed on initially included studies to identify additional studies. In addition, a PubMed citation search was conducted on all studies included in the final review.

### Identification of studies

The titles of the studies generated from the searches were reviewed for inclusion by one author alone. Abstracts from potentially relevant titles were then reviewed against the inclusion and exclusion criteria by two authors. The full texts of articles were obtained for all abstracts deemed to be potentially relevant and were reviewed by three authors. Systematic reviews identified by the search were scrutinised by one author for additional studies. Any disagreements were resolved by discussion and consensus reached before final inclusion. The study selection process from identification to exclusion was documented using the PRISMA flow chart.

### Quality assessment of evidence

The Cochrane Collaboration’s recommended tool for assessing risk of bias was used to assess the risk of bias (35). The potential sources of bias assessed were random sequence generation (selection bias), allocation concealment, blinding of participants and personnel (assessment was made for each) and outcome assessment (detection bias due to knowledge of the allocated interventions), incomplete outcome data, selective outcome reporting and other sources of bias. The studies were classified as containing high, low or unclear risk of bias for each of the criteria for judging risk of bias, and a conclusion regarding the overall risk of bias was made. Classification was based on the judgement of three authors following the guidelines outlined in the Cochrane Handbook.

### Data extraction and description

Outcome measures sought in the publications were defined as either ‘critical’ or ‘important’ according to GRADE (36) (see Table 1). Data were described in the text. Since it was not expected that the included studies would be judged to be clinically homogeneous, no meta-analysis was performed. In cases where statistically significant effects were demonstrated, effect sizes (Cohen’s d) of the study interventions were either obtained from the publications when provided or calculated for this review. Cohen proposed interpreting *d* = 0.2 as a small effect size, *d* = 0.5 as a moderate effect size and *d* = 0.8 as a large effect size (37, 38).

## Results

### Articles selected for review

**Fig. 1:**
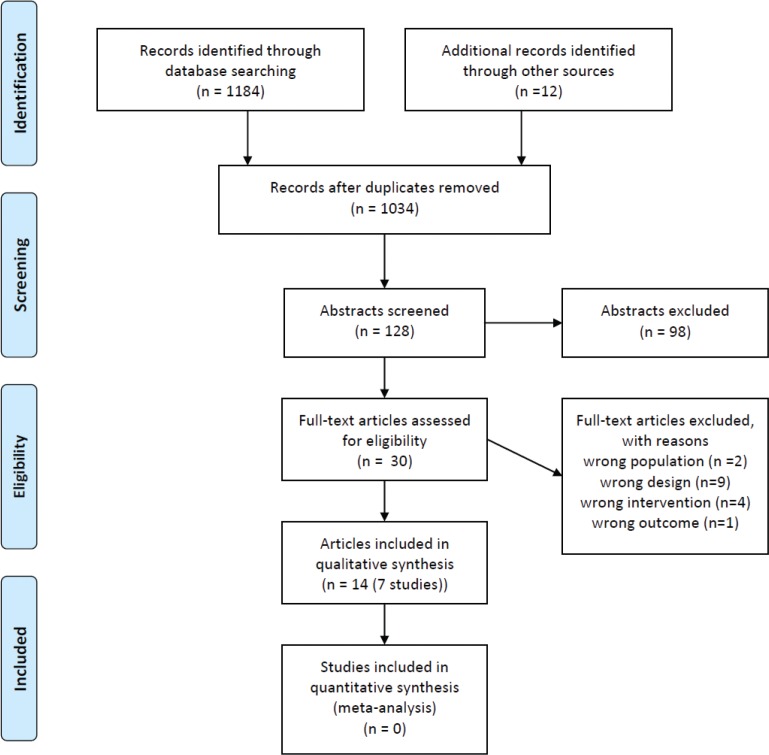
Search strategy, study selection and process of identification of suitable studies.

The initial searches resulted in 1,196 titles. Screening of titles identified 128 abstracts for further assessment. A total of 30 full-text articles were reviewed. Seven studies (reports published in 14 papers) fulfilled the inclusion criteria for the present review (see flow diagram in Fig. 1): Härmä et al. (39, 40), the POWER study (41, 42), Guillermard et al. (43), Lim et al. (44) and the PHLAME study (45–49), including a pilot study (50). Two additional relevant studies were identified in the updated search: Leedo et al. (51) and Matsugaki et al. (52). A list of the excluded full-text articles is available from the last author.

### Details of the studies

The studies had been performed in different parts of the world, including East Asia, Europe, the United States and Australia (Table 2). The majority of studies were performed in the health care and manufacturing sector among shift workers including nurses and other health care workers (39, 40, 51, 52), plant workers (41, 42), fire fighters (45–50) and different shift workers, including police officers and nurses (43). One study did not report the occupation of the night shift workers (44).

**Table 2:** Description of the included studies

*Population (P):* People working irregular hours, meaning ‘around the clock’
*Intervention (I):* Healthy working environment
*Comparison (C):* Usual care
*Outcomes (O):* Critical: Dietary and physical activity habits Important: General well-being, quality of life, sleep circadian rhythm, cognitive performance, psychological stress, blood measurements, body composition, muscle strength, influence on work performance, adverse events, dropouts
*Study design:* Randomised controlled trials, cluster-randomised controlled trials and randomised crossover studies

The number of participants within each study was generally small with four studies having between 30 and 75 participants (39, 40, 44, 50–52), while three studies had a larger sample size of between 110 and 1,000 participants (41–43, 45). In all, the participants were young or middle-aged adults. In three studies, the target population included only men or consisted predominantly of male employees (41, 42, 44, 45, 50), two included only women (39, 40, 52) and two studies included both male and female employees (43, 51). The length of the interventions varied from 2 to 12 months.

### Interventions

*A broader lifestyle intervention approach* was reported in two studies; the POWER (41, 42) and the PHLAME study (45–50). The POWER study (41, 42) involved a 3-month cluster randomised controlled trial among 110 male overweight/obese aluminium plant workers in Australia and focused on weight loss. It included an information session during work, a handbook with provision of information regarding the programme, a study website including a tutorial and a user guide, seven individualised dietary feedback sheets, group-based financial incentives and a pedometer as part of a group-based, cognitive theory-guided weight-loss programme versus usual activity (41). In the PHLAME cluster randomised controlled trial (45–48, 50), two types of intervention among firefighters during duty hours were compared with a control group in a 6-month pilot study with 33 participating firefighters and in a 12-month main study (followed by a scaled-down booster programme the following year and a 4-year follow-up period) with 397 participating firefighters. The first type of intervention involved a *team-centred curriculum* with a group-designated team leader, team leaders’ manual, workbooks, lesson plans and team leaders’ orientation focusing on healthy nutrition, physical activity and energy balance plus additional core topics, such as stress or sleep. The second type utilised *individual-centred motivational interviewing* with a counsellor to discuss and review the goals, values and priorities for change in firefighters’ personal lifestyle behaviours, plus a short follow-up option in person or via phone (45). Further, a retrospective follow-up study over a 6-year period was conducted among the firefighters in the PHLAME study to evaluate medical costs among the fire departments participating in the health promotion intervention department compared with other fire departments (49).

*Physical activity* was the focus of the intervention in three studies (39, 40, 44, 52). In the randomised controlled trial by Härmä et al. (39, 40), a 4-month physical training programme was individually tailored for 75 female nurses in Finland according to their submaximal ergometer test, age and sports habits. In the exercise programme, both circulatory and musculoskeletal systems were activated by jogging, running, swimming, skiing, walking and gymnastics in 2–6 training sessions per week, according to the physical condition of the subject. In a 10-week randomised controlled trial by Lim et al. (44), 30 male night shift workers in South Korea were instructed to include three 10-min walking exercise sessions on 3 days per week into their night routines while at work. Based on the highest heart rate recorded during the VO_2_ max test, a target zone of the maximal heart rate was established for each subject. The exercise programme was self-monitored, and participants were encouraged to schedule their walking time into their night routines while at work. In a randomised controlled trial by Matsugaki et al. (52), the 12-week exercise programme intervention among 30 female nurses conducting shift work consisted of either exercise under the individual supervision of a physical therapist or voluntary exercise without supervision at the hospital.

*Offering healthier food or meal options* was the focus of the intervention in two studies. Guillermard et al. (43) tested in a randomised controlled trial the effect of offering a fermented dairy product containing *Lactobacillus casei* twice a day as a breakfast and dinner supplement in a placebo-controlled study of 1,000 male and female shift workers in France. This was a 3-month study with 1-month follow-up aiming at reducing the risk of common infections. Leedo et al. (51) tested in a randomised crossover controlled study the effect of 8 weeks of increased availability of healthy meals at work, including a daily cold lunch meal, bottled water and a snack among a total of 59 hospital health care employees, including 16 employees working in shifts.

Additional information about the included studies, that is, the outcomes assessed and significant effects, can be found in Table 2.

### Study quality

Three of the studies, Guillermard et al. (43), the POWER study (41, 42) and Leedo et al. (51), were assessed as having a low risk of bias (see Table 2). This includes also the risk of carry-over effect in the crossover study by Leedo et al. (51), based upon the report that the researchers had examined the possible existence of a carry-over effect of treatment sequence on all outcomes. However, the study by Leedo et al. (51) was assessed as having an unclear to high level of bias in the group of shift workers as a subsample within the larger study sample due to small number of subjects. Four studies were assessed as having unclear or high risk of bias, which was mainly due to lack of blinding of participants, personnel or outcome assessment. In these studies, the outcomes may have become influenced by lack of blinding of the allocated interventions and the use of subjective and self-reported outcomes (39, 44, 45, 50, 52), other sources of bias, for example, an imbalance in baseline characteristics (44, 45, 50), a high dropout rate (39, 40) or in situations where the influence of clusters (in the analysis) had not been considered (45, 50).

### Critical outcomes

*Dietary habits* were evaluated in the PHLAME study (45, 50), the POWER study (41) and the study by Leedo et al. (51) among firefighters, aluminium plant workers and health care staff, respectively. All three found some significant improvement in dietary behaviour, for example, an increased intake of fruit and vegetables in the PHLAME main study (effect sizes: 0.2 and 0.4 in the individual and team-based groups, respectively) (45), a decreased intake of sweetened beverages in the POWER study (effect size: 0.5 to 0.6) (41) and an increased intake of water among the shift-work subgroup in the study by Leedo et al. when comparing intervention versus control group (not possible to calculate effect size) (51). On the contrary, no intervention effect on fruit and vegetable intake was found in the POWER study (41). PHLAME and POWER studies both used a questionnaire to assess dietary habits, whereas in the study by Leedo et al. (51) participants completed a 4-day self-reported dietary record. In the PHLAME study, mediating factors for the improvement in fruit and vegetable intake included an increased knowledge of the benefits of fruit and vegetable intake (significant for fruit intake) and an improved level of social support (dietary norms) experienced from co-workers (significant for vegetable intake) (45, 47).

*Physical activity* measures included changes in physical activity habits and maximum oxygen uptake and were assessed in four studies (39, 41, 45, 50, 52). All four, expect for the PHLAME main study, found a significant impact on either maximal oxygen uptake (VO_2_ max) (39, 52) or physical activity habits assessed via questionnaire (41, 50). In the study by Härmä et al., the physical exercise intervention resulted in an improved VO_2_ max among nurses (effect size: 0.4) (39). Similarly, in the study reported by Matsugaki et al. (52), VO_2_ max increased significantly in the group receiving exercise supervision (effect size: 0.6) compared with the voluntary exercise group. Contrary to these, the PHLAME lifestyle interventions among firefighters resulted in non-significant impact on VO_2_ max in the pilot study (46) and in the main study (41). However, the level of physical activity, assessed via questionnaire, was reported to have significantly improved in the team-based group in the PHLAME pilot study (effect size: 1.1) (46).

*Physical strength* was assessed in three studies (39, 45, 52), and a significant impact was reported in all on either number of sit-ups or on muscle strength measured by strength of the knee extensor muscle. Number of sit-ups from baseline to post-intervention increased significantly both in the PHLAME main lifestyle intervention (not possible to calculate effect size) (45) and in the nurses’ physical activity study by Härmä et al. (39) (effect size: 0.9). Also, the Matsugaki et al. study (52) reported significant improvement in muscle strength (180 deg/sec KET) among nurses in the supervised exercise group over time (effect size: 1.3).

*Long-term change in diet and physical activity habits* were assessed in the PHLAME study, which included a 4-year follow-up of their participants. The authors concluded that 1-year effects of the programme did not remain over time compared to the control group, but the long-term pattern of behaviours in both groups suggested that the worksites, as a whole, had continued to improve in outcome measures for several years following the programme (47).

### Important outcomes

*General well-being including quality of life* was assessed in three studies via questionnaires (42, 43, 45). Two of the interventions showed significant improvements in either overall well-being or quality of life using different scales. In the PHLAME study, the index of general well-being improved significantly in both types of lifestyle intervention (individual- and team-based) among firefighters (45). The POWER trial used the SF-12 questionnaire and found a significant positive effect of the broader lifestyle intervention on mental health (effect size: 0.7) but not on physical health among aluminium plant workers (42). However, no difference was found by Guillermard et al., who used the original larger SF-36 scale in their study on offering a fermented dairy product as a supplement to reduce the risk of common infections among shift workers (43).

*Sleep circadian rhythm* was examined in two studies. Härmä et al. found a significant positive effect of physical exercise on sleep length and reduction of fatigue among nurses (not possible to calculate effect size) (39), while the POWER study (42) did not observe any significant benefits in sleepiness score of a broader lifestyle intervention for weight loss among aluminium plant workers.

*Psychological stress* was measured in several ways: via blood pressure, heart rate, testosterone level and body temperature in a total of four studies (39–41, 43, 44). Two of them found a significant positive effect on blood pressure and/or heart rate (39, 41), while two did not (43, 44). Morgan et al. (41) found a positive effect of the POWER lifestyle intervention on systolic blood pressure among aluminium plant workers in their group-based weight loss programme compared to control (effect size: 0.5). Psychological stress was assessed via heart rate in three studies. Two of these found a significant positive effect: Härmä et al. (39) offering physical exercise to female nurses (effect size: −0.4) and Morgan et al. (41) offering a lifestyle intervention to aluminium plant workers (effect size: −0.8). In contrast, no difference between intervention and control groups was reported in the study by Guillermard et al. offering two daily fermented dairy products to male and female shift workers (43). Härmä et al. (40) also measured psychological stress by means of body temperature (40) and did not observe any benefit of the intervention in nurses’ physical exercise intervention.

*Cognitive performance* was evaluated in three studies using different measures. In two, positive effects were found in cognitive performance measures (40, 51). The study by Härmä et al. found a significant positive effect of physical exercise on alertness in nurses during the night shift (not possible to calculate effect size) (40), and the study by Leedo et al. (51) found a significant positive effect on total mood-related score among the subgroup of shift workers (effect size: 0.3). In the latter study, however, no effect on reaction time was found. Contrary to Leedo et al.’s study (48), Matsugaki et al.’s study did not find any significant effect in total mood-related score among nurses (52).

*Blood measures*, for example, cholesterol and the biomarker cathepsin, were evaluated in three studies (44, 50, 52). All three found significant positive effects: decreased level of LDL-cholesterol in the PHLAME pilot study in both the team- and individual-based groups (effect sizes: −0.2 and −0.4, respectively) (50), increased level of HDL cholesterol among nurses in the supervision exercise group in the study by Matsugaki et al. (effect size: 0.2) (52) and a decrease in cathepsin S and L in the Lim et al.’s study on night shift workers in the exercise intervention group compared to control (effect size: −0.4) (44).

*Body composition* was assessed in all studies except the Leedo et al.’s study (51). Two studies assessing change in lean body mass (LBM) in a physical exercise intervention among night shift workers did not find any effect (44, 52). However, one of these studies found a small but significant positive effect on percentage of body fat (effect size: −0.03) (44), while no difference was observed in the others (39, 45, 52). Finally, Morgan et al. (42) assessed waist circumference and found a positive effect of the intervention among the aluminium plant workers (effect size: −0.6).

*Weight and body mass index (BMI)* were measured in all studies except the one by Guillemard et al. (43). They reported either significant difference in weight loss between intervention and control groups (41, 44), no significant weight changes compared to control group or control period (39, 51, 52), or less weight gain in the intervention group than the control group (45). The POWER study resulted in both significant weight loss and improved BMI among overweight plant workers in the intervention group compared to control (effect sizes: −0.3 and −0.4, respectively) (41). The goal of the PHLAME study was, among others, to improve energy balance among firefighters. This was succeeded during the 1-year intervention, where both the team- and individual-based groups gained less weight than the control group (45). The three studies testing the effect of physical exercise (39, 40, 44, 52) measured weight, and in two, the normal weight participants in the intervention group had a small loss of weight which was more than in the control group, but this type of unintentional weight loss was reported as significant only in the study among night shift workers in the study by Lim et al. (effect size: −0.1) (44). In the study by Leedo et al., the participants kept a stable weight throughout the intervention offering foods and beverages to improve overall dietary intake (51).

*Influence on work performance* was assessed in three studies (41, 43, 46, 49), all with positive outcomes. The POWER study among plant workers (42) assessed work place productivity, injuries at work and absenteeism and found a positive effect on all these outcomes (effect sizes: 0.5 to 0.7). In the retrospective PHLAME follow-up study, a reduction in compensation claims and medical costs was seen (49). Guillermard et al. (43) found a positive significant effect from offering fermented dairy products among shift workers’ cumulated days with fever (effect sizes: −1.2) compared to control (only for the whole study phase) but not on the primary outcome, that is, cumulated time with chronic infectious diseases.

*Adverse effects* were only assessed in two studies. Guillermard et al. (43) assessed blood pressure, heart rate and weight, and Matsugaki et al. assessed muscle pain and physical fatigue (52). They did not find any difference between study groups.

## Discussion

The present review carried out a systematic identification, analysis and quality assessment of the evidence on the impact of workplace interventions to promote healthy food and/or physical activity on dietary and physical activity outcome measures among people working ‘around the clock’ compared with controls receiving usual care. A total of seven studies (reported in 14 papers) were included in the final analysis: two having a broader lifestyle approach, three based on physical exercise and two based on offering healthy meals as a replacement of ordinary meals and offering food supplements to existing workplace meals. In general, a positive effect was seen on several of the outcomes assessed irrespective of the intervention approach.

Taken together, the studies showed small-to-moderate effect sizes on several measures, including dietary and/or physical activity measures, suggesting acceptable effectiveness for interventions involving community-level behaviour change. This review showed moderate positive effects on several critical and important outcomes in the two larger studies with a broader lifestyle approach focusing on dietary habits among aluminium plant workers (the POWER study) (41, 42) and on physical activity among firefighters (the PHLAME study) (45–50). These outcomes included improvements in intake of fruit and vegetables, intake of sweetened drinks, weight status, physical activity, strength and work performance. Moderate positive effects were seen also in the two smaller ‘high-intensity’ studies using individually tailored or supervised exercise programmes among nurses (39, 40, 52). Here, the outcomes included improvements in physical activity, strength and LDL-cholesterol levels. Small-to-moderate positive effects were seen in the intervention in which a ‘one type fits all’ exercise programme was added to the routines of night shift workers by Lim et al. (44). Also, in general, limited effects were reported in the study in which a food supplement was offered (43). The outcomes in these studies included improvements in weight status and the biomarker cathepsin in the former, and improvements in incidence of respiratory and gastrointestinal common infectious diseases and work performance in the latter study. Small effects were also shown in the study by Leedo et al. (51), providing health workers with healthy foods and beverages during working hours. This study reported some positive results, including an increase in water intake but did not reach its targets in increasing overall dietary intake, for example, an increase in fruit and vegetable intake. The Leedo et al.’s (51) study outcomes might have shown more effect, had the sample size been larger in terms of the number of shift workers. In light of our literature review, it may be prudent to say that the limited outcomes in these three studies (43, 44, 51) may at least partly be attributable to a lack of use of participatory and empowerment strategies to assure that the intervention content is responsive to the employees’ needs and priorities. The unique challenges encountered by shift workers in adhering to a healthy diet are important and should be acknowledged in successful intervention design (41).

In a similar vein, a review by Verweij et al. of worksite interventions, not limited to shift workers, to promote physical activity and dietary behaviour, showed greater effect, for example, success in weight loss, in interventions which contained environment components besides personal components (32). On the contrary, the review by Allan et al. (27) showed that significant change in primary outcome measures of eating behaviours, for example, increased fruit and vegetable consumption, was reported only by about half of the identified worksite interventions that had used solely environmental strategies to alter eating behaviours. Implementing environmental interventions can, according to Tam et al. (31) in their review of long-term effectiveness of work-based lifestyle interventions to tackle overweight and obesity, prove problematic due to the multiple layers of commitment needed at different levels of the organization, for example, support at the management level and on behalf of the participants to ensure sufficient individual participation. Our review concurs with the conclusion of Tam et al. (31) that the most effective interventions may be the ones that are of high intensity or include a specific motivational component besides interventions with multiple lifestyle components.

To conclude, the findings from our review have highlighted a lack of evidence from workplace interventions to promote healthy food and physical activity during working hours among people who work during unorthodox hours, ‘around the clock’.

### Quality of the studies reviewed

Several of the studies were assessed as having an overall high risk of bias with regard to the outcomes (see Table 2). It is important to note that it has probably not been feasible to conduct blinding in terms of participants and staff in the type of interventions studied, that is, focusing on diet or physical activity in real-life settings. Achieving a high-quality rating was especially problematic because of the subjective outcomes (e.g. quality of life) and self-reported measures (via questionnaires) used in many of the reviewed studies in assessing their effect. In future studies in this field, it is, therefore, important to consider more objective outcome parameters. Further, offering an active control condition, as opposed to no treatment control, may facilitate blinding (53). Another major issue possibly contributing to bias in the assessment of outcomes common in the now reviewed studies was an imbalance in baseline characteristics. This may, in turn, have contributed to the lack of effect on some of the outcomes. In addition, only three studies identified primary outcome and presented a power calculation, which reduces their comparability and contribution to evidence base in this field.

### Limitations and strengths of the systematic review

With our search terms, we identified only seven studies that could be included in the analysis. This is an indication of a lack of studies on the promotion of physical activity and dietary lifestyle changes among employees working ‘around the clock’. Due to the small number of studies, a wide mixture of approaches (from broader lifestyle intervention to food supplements), the generally small sample sizes, the variation in intervention duration and the different kind of shift work occupations and settings, we were only able to perform a qualitative extract of main patterns in the effectiveness of the different types of intervention. A higher number of larger well-focused studies are needed to compare different approaches in different types of shift work under irregular working hours and among different occupational groups. For example, the two studies focusing specifically on male shift workers were the ones that employed a broader lifestyle approach and showed evidence of effectiveness. It would be of interest to replicate such an approach among both female and male shift workers and different occupational groups. Women have been frequently reported to engage in far more health-promoting behaviours than men and to obtain healthier lifestyle patterns (54). On the contrary, from a behavioural standpoint, some evidence suggests that men may engage better with a lifestyle programme once committed, although getting them to initially commit might be more challenging (55). A further limitation is the clinical inhomogeneity in the included studies which did not warrant a meta-analysis but would have rendered it meaningless. Finally, a limitation of the search strategy was that articles not published in English were not included and moreover that the updated search in October 2017 was restricted to Google Scholar and PubMed, which may mean that some relevant studies were missed.

The main strengths of this review were that it employed a comprehensive search strategy and brought together research findings on the impact of worksite interventions to promote healthier food and/or physical activity among an understudied but critical group of employees who work ‘around the clock’. Despite the fact that the interventions were focused on healthy food and/or physical activities, other beneficial outcomes were measured such as work performance. Of course, a causal relation between diet/physical activity and the important outcomes can be questioned. According to our knowledge, no other systematic review has considered employees working ‘around the clock’. Neil-Sztramko et al. (56) has critically reviewed the literature of worksite health-related interventions to prevent negative health effects among shift workers but limited their target group to night shift workers and only two of the 38 studies reviewed had a focus on healthy food or physical activity. These two studies (39–42) have been included in the present review.

### Implications for health promotion programming and practice

Our review highlights the need for further testing of the broader lifestyle interventions as well as the individualised and high-intensity approaches in the target population. This arises from the two interventions carried out among male participants in the PHLAME study (45–50) and the POWER study (41, 42), and the two physical exercise studies among nurses (39, 40, 52). These studies included some degree of participatory and empowerment strategies, approaches that have been shown to be important in assuring programme responsiveness to employees’ needs and priorities (3, 26). The long-term follow-up of the PHLAME study suggested that the participating worksites, as a whole, including the control participants, continued to improve in their outcome measures for several years following the programme (47). This provides further encouragement for knowledge translation into practice. The review also provides some support for improving the food and physical activity environmental strategies within organisations, including the provision of healthy meals and beverages, as in the Leedo et al.’s study (51). However, the study population (51) was too small to make firm conclusions.

In future studies, it is necessary to tailor intervention studies with respect to work schedules, meal breaks and mobile or mixed work places. The special challenges with respect to working irregular hours and circadian stress need to be addressed, that is, to recommend a healthy timing of eating with respect to circadian rhythm factors. Although there are still unsolved issues regarding the association between shift work and disease, we have enough knowledge to prompt preventative action (57).

### Future perspectives

This review highlights the need for more evidence on the effectiveness of workplace interventions to promote healthy food and physical activity among people working irregular or extended hours ‘around the clock’. Future research could focus on the nutritional and social aspects regarding eating behaviour in this target population, for example, to describe the effect of work schedule on dietary intake and meal timing, and the strategies people use in relation to their food and eating during irregular or extended working hours.

In particular, more knowledge is needed on the coping strategies and interventions to support shift workers employed in the retail and service sector in our modern ‘24-hour society’. This was a sector from which no studies were identified in this review. More research is also needed on effective approaches to promote health and well-being at any age by adopting a life-course approach and including older workers in the studies (58).

## Appendix A

PubMed Search Terms for the Literature Search

( ( ( ( ( ( ( ( (‘physical activity’[All Fields]) OR (‘exercise’[MeSH Terms] OR ‘exercise’[All Fields])) OR (‘exercise therapy’[MeSH Terms] OR (‘exercise’[All Fields] AND ‘therapy’[All Fields]) OR ‘exercise therapy’[All Fields])) OR ( (‘diet’[MeSH Terms] OR ‘diet’[All Fields] OR ‘dietary’[All Fields]) AND (‘behaviour’[All Fields] OR ‘behaviour’[All Fields]))) OR (‘meals’[MeSH Terms] OR ‘meals’[All Fields])) OR (‘overweight’[MeSH Terms] OR ‘overweight’[All Fields])) OR (‘counselling’[All Fields] OR ‘counseling’[MeSH Terms] OR ‘counseling’[All Fields])) OR (‘policy making’[MeSH Terms] OR (‘policy’[All Fields] AND ‘making’[All Fields]) OR ‘policy making’[All Fields])))

AND

(shift work* OR night work* OR evening work* OR work hour*)
